# Heat stress response mechanisms in pollen development

**DOI:** 10.1111/nph.17380

**Published:** 2021-05-20

**Authors:** Palak Chaturvedi, Anna J. Wiese, Arindam Ghatak, Lenka Záveská Drábková, Wolfram Weckwerth, David Honys

**Affiliations:** ^1^ Molecular Systems Biology (MOSYS) Department of Functional and Evolutionary Ecology Faculty of Life Sciences University of Vienna Althanstrasse 14 Vienna 1090 Austria; ^2^ Laboratory of Pollen Biology Institute of Experimental Botany of the Czech Academy of Sciences Rozvojová 263 Prague 6 165 02 Czech Republic; ^3^ Vienna Metabolomics Center (VIME) University of Vienna Althanstrasse 14 Vienna 1090 Austria

**Keywords:** heat stress (HS), heat stress response (HSR), multiomics, pollen development, thermotolerance

## Abstract

Being rooted in place, plants are faced with the challenge of responding to unfavourable local conditions. One such condition, heat stress, contributes massively to crop losses globally. Heatwaves are predicted to increase, and it is of vital importance to generate crops that are tolerant to not only heat stress but also to several other abiotic stresses (e.g. drought stress, salinity stress) to ensure that global food security is protected. A better understanding of the molecular mechanisms that underlie the temperature stress response in pollen will be a significant step towards developing effective breeding strategies for high and stable production in crop plants. While most studies have focused on the vegetative phase of plant growth to understand heat stress tolerance, it is the reproductive phase that requires more attention as it is more sensitive to elevated temperatures. Every phase of reproductive development is affected by environmental challenges, including pollen and ovule development, pollen tube growth, male–female cross‐talk, fertilization, and embryo development. In this review we summarize how pollen is affected by heat stress and the molecular mechanisms employed during the stress period, as revealed by classical and ‐omics experiments.

## Introduction

Due to global warming, heatwaves are predicted to increase in many regions across the globe, posing a massive threat to agricultural security. Elevated temperatures, whether transient or constant, have an adverse impact on crop yields. Such conditions bring about changes in plant morphology, physiology, and biochemistry, which in turn negatively impact plant growth and development (Begcy & Dresselhaus, 2018). Extreme heatwaves are not solely responsible for these adverse impacts, as it has been shown that even mild temperature fluctuations have an impact. For example, it was found that for every 1°C increase in growing‐season minimum temperature, rice grain yields declined by 10% (Peng *et al*., 2004). Similarly, for every 1°C increase in temperature above 21°C in the day and 16°C at night, wheat (*Triticum aestivum*) yields declined by 5% (Tashiro & Wardlaw, 1989), while every day spent above 30°C resulted in a 1% decline in maize yields (Lobell *et al*., 2011).

Increased temperatures can often have a detrimental effect on plant sexual reproduction, which can lead to a reduction in the fertility of many species. Sexual reproduction in angiosperms comprises three phases: gametophyte development, the progamic phase, and embryo and seed development (from zygote to seed). The male gametophyte (pollen) plays a key role in plant reproduction and crop productivity, through the formation and delivery of male sperm cells to the female gametophyte for double fertilization (Carrizo García *et al*., 2017). Heat stress (HS) affects male and female gametophytes differently. Male gametophytes are more sensitive to HS throughout their development (Zinn *et al*., 2010; Hedhly, 2011); it affects pollen quantity and morphology, the architecture of cell walls and, importantly, pollen metabolism (Hedhly, 2011). However, sensitivity to HS varies between species when the high temperature modes are applied (Parrotta *et al*., 2016).

The heat stress response (HSR) of sporophytic tissues has been the subject of a plethora of studies; however, these research findings are not relevant to pollen, as it has evolved a distinct and rather complex HSR compared to that of sporophytes (Bokszczanin *et al*., 2013). This alone justifies the need for pollen‐specific HSR studies (Mesihovic *et al*., 2016). Due to advances in high‐throughput profiling methods and technologies related to pollen isolation and separation, our understanding of the HSR during the course of pollen development has dramatically improved. In this review we summarize what is known about the effects of HS on pollen development and the pollen HSR during various stages of development, using studies that utilized classical and multiomics approaches.

## Cytological alteration during pollen development and pollen tube growth under heat stress

Plants experience heat stress when temperatures exceed a certain threshold (i.e. 5–10°C above their optimal growth temperatures; Kranner *et al*., 2010), with their response depending on the duration and intensity of the stress (Larkindale *et al*., 2005). The primary response, however, involves massive transcriptional and translational changes. Pollen development, specifically, represents a very narrow developmental window. It is a complex process that requires the coordinated activity of different gametophytic and sporophytic cell types and tissues (Hafidh *et al*., 2016), making it particularly sensitive to environmental challenges. The findings of a study using crosses of tomato (*Solanum lycopersicum*) and *Brassica napus* plants, with male and female reproductive organs independently subjected to various heat stresses, suggested that when high temperature stress is applied separately to male and female gametes before pollination, pollen represents the weakest link (Zinn *et al*., 2010). Hence, when HS is applied during this developmental window, it can potentially disturb reproductive development, leading to pollen abortion, which hampers the fertilization process.

### Effects of temperature stress on pollen development and pollen tube growth

Within anthers, diploid pollen mother cells (microsporocytes) undergo meiosis to give rise to four haploid microspores held together in the tetrad by a thick callose wall. The callose wall later gets degraded by the enzyme callase (secreted by the tapetum), releasing the individual microspores. From here, microspores increase in size and vacuolize, and their nuclei migrate to the periphery. The polarized microspores then undergo a highly asymmetric mitotic division (pollen mitosis I, PMI) (Hafidh *et al*., 2016) to form a large vegetative cell and a small generative cell. The microspore is thus the pluripotent initial of the male germline that establishes cells with two different fates (Berger & Twell, 2011). The germ cell undergoes one more round of mitotic division (pollen mitosis II, PMII), to produce the two sperm cells required for double fertilization. Mature pollen is shed from the anthers in either a bicellular or tricellular form, depending on whether PMII occurs before pollen maturation or after pollen germination.

**Fig. 1:**
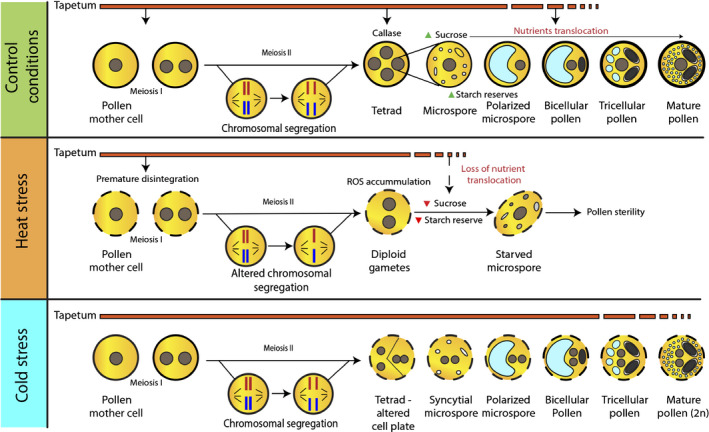
Schematic overview of cytological alterations imposed by heat and cold stress during male gametophyte development. Under heat stress conditions, the tapetum starts to degrade prematurely, which affects the nutritional supply to the developing pollen and leads to pollen sterility. Furthermore, the concentration of the soluble carbohydrate and starch reserves decreases in the developing anthers, followed by an increase in the reactive oxygen species (ROS) accumulation. Under cold stress, the tapetum does not undergo early degradation but rather shows abnormal expansion at the microspore stage and persists until pollen maturity. Cold treatment has a restitutive effect on the male meiosis; it significantly alters cell plate expansion and cell wall formation during meiotic division. Furthermore, cold‐stressed pollen mother cells produce microspores harbouring multiple haploid nuclei. Before pollen mitosis I (PMI), these nuclei fuse and develop into diploid or polyploid pollen.

Successful and coordinated pollination and fertilization require the synchronous development of microspores within an anther. This process is controlled at several check‐points, and when it fails (e.g. due to stress), developmental asynchrony promotes physiological and metabolic differences among microspores (Fig. 1) (Giorno *et al*., 2013). It subsequently increases their competition, in terms of resources during development, water for rehydration on the stigma, and pollen tube growth. In most plants, the onset of meiosis and microspore development towards PMI seem to be the processes that are most sensitive to environmental stress conditions (De Storme & Geelen, 2014; Muller & Rieu, 2016; Rieu *et al*., 2017; Begcy *et al*., 2019). Indeed, the earliest heat‐induced defects in *Arabidopsis* male gametophyte development occur during meiosis, through an increased frequency of crossing over and homologous recombination (Boyko *et al*., 2005; Francis *et al*., 2007). Moreover, exposure of *Arabidopsis* and rose (*Rosa* spp.) plants to mild HS (e.g. 48 h at 36°C) results in meiotically restituted dyads and triads that contain unreduced, diploid male gametes instead of the standard haploid ones (Pecrix *et al*., 2011; De Storme & Geelen, 2020). In addition to affecting meiosis, HS also affects cytoskeletal dynamics and spindle orientation in *Arabidopsis* and tobacco (*Nicotiana tabacum*; De Storme & Geelen, 2013; Parrotta *et al*., 2016). In the context of changes to the secondary metabolome, HS brings about an increase in flavonoid abundance in polarized microspores in tomato (Paupière *et al*., 2017a). Flavonoids play an important role in reactive oxygen species (ROS) detoxification (Rice‐Evans *et al*., 1996). Moreover, the abundance of conjugated polyamines is 37% lower in late pollen developmental stages compared with polarized microspores following HS (Paupière *et al*., 2017a). Polyamines increase the activity of antioxidant enzymes, which play a role in ROS detoxification (Chen *et al*., 2018).

Heat stress also affects the cell layers that surround microsporocytes. In common bean, (*Phaseolus vulgaris*) linear, looped, wavy or circular endoplasmic reticulum (ER) structure has been observed in tapetal cells following HS, instead of the customarily stacked rough ER observed under nonstressed conditions (Suzuki *et al*., 2001). Aberrations in tapetal development caused by HS have been reported in barley (*Hordeum vulgare*), cowpea (*Vigna unguiculata*), wheat and stiff brome (*Brachypodium distachyon*); (Saini *et al*., 1984; Ahmed *et al*., 1992; Abiko *et al*., 2005; Oshino *et al*., 2007; Sakata *et al*., 2010; Harsant *et al*., 2013). Heat stress also brings about premature degeneration of the tapetum, a layer of nutritive cells responsible for the nutrition of developing pollen grains. The tapetum is very rich in mitochondria compared to vegetative tissues (Lee & Warmke, 1979; Selinski & Scheibe, 2014). Under HS, the vast number of mitochondria most likely contribute to a dramatic rise in ROS generation as by‐product of aerobic metabolism (Mittler, 2017), and when vast amounts accumulate due to stress, they cause oxidative damage and cell death (Sharma *et al*., 2012). Reactive oxygen species signaling plays an important role in developmental programmed cell death (PCD) of the tapetum in dicot species such as *Arabidopsis,* tomato and tobacco, and in monocot species such as rice (*Oryza sativa*; Hu *et al*., 2011; Yu *et al*., 2017). However, the failure or premature PCD of the tapetum brings about male sterility (Kurusu & Kuchitsu, 2017). Indeed, during HS, ROS accumulates in anthers, causing an imbalance between ROS levels and ROS quenching enzymes, which induces premature PCD and degradation of the tapetal cell layer (Fig. 1) (Zhao *et al*., 2018).

However, during HS, ROS also participate in signaling cascades that produce detoxification enzymes (e.g. ascorbate peroxidase and catalase) that function to lower the amount of ROS in the cell, thereby forming a regulatory loop mechanism (Chaturvedi *et al*., 2013; Guan *et al*., 2013; Qu *et al*., 2013). This has been demonstrated in the pollen of wheat (Kumar *et al*., 2014) and *Sorghum bicolor* (Djanaguiraman *et al*., 2014), where an increase in ROS levels was accompanied by an increase in the abundance of detoxification enzymes. Finally, ROS accumulation has been shown to induce the expression of HEAT SHOCK TRANSCRIPTION FACTOR A1 (HsfA1), which stimulates HS‐responsive gene expression (Yoshida *et al*., 2011; Guan *et al*., 2013). Under cold stress conditions, however, the tapetum does not show early abortion, but rather shows abnormal expansion at the microspore stage (Oda *et al*., 2010) and persistence until pollen maturity stage (Fig. 1), a phenomenon observed in wheat, barley (*Hordeum vulgare*), stiff brome and rice (Oda *et al*., 2010; De Storme & Geelen, 2014; Muller & Rieu, 2016).

The functional, progamic phase commences once pollen reaches the stigma. Following rehydration on the stigma, the pollen grain germinates and produces a pollen tube that penetrates the pistil. Pollen tubes are highly specialized structures that deliver male gametes to the embryo sac for double fertilization (Hafidh *et al*., 2016). Pollen tube growth is an energetically demanding process that is dependent on the sufficient building and utilization of reserves, fast cell wall synthesis and adequate cell–cell communication. Mitochondrial decay under high temperatures causes defects in supporting these processes, as observed in rice pollen (Khatun & Flowers, 1995) and cultured tomato pollen tubes (Karapanos *et al*., 2009). At the ultrastructural level, HS induces changes in the isoform content and distribution of cytoskeletal subunits in tobacco pollen tubes, affecting the accumulation of secretory vesicles and the distribution of cellulose and callose synthases, enzymes involved in cell wall synthesis (Parrotta *et al*., 2016). Other effects of HS on the progamic phase include a decrease in ovule viability, altered stigma and style position, and a loss of stigma receptivity, which leads to impaired fertilization (Foolad & Sharma, 2005; Kumar *et al*., 2013; Gupta *et al*., 2015).

## Stress sensing – setting in motion the heat stress response (HSR)

In order to respond to stress, plants first need to perceive it. Plant cells can sense stress at several interfaces using specific sensors. These include phospholipid membranes (due to their fluidity and permeability), Ca^2+^ flux, protein stability, the unfolded protein response (UPR) in the ER and cytoplasm, chromatin status and histone modifications, enzymatic reactions, and mRNA structure and stability. When stimulated, these pathways trigger signal transduction cascades that bring about the HSR, which functions to restore cellular homeostasis (Mittler *et al*., 2012; Zhu, 2016).

**Fig. 2:**
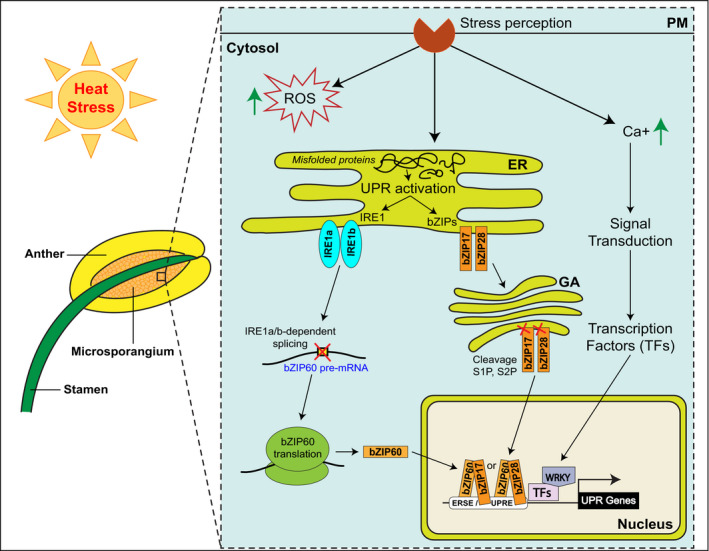
Heat stress sensing and response mechanism during male gametophyte development. Elevated temperature stress is perceived by the pollen vegetative cell, which triggers Ca^2+^ flux, ROS accumulation in the cytosol and activation of the unfolded protein response (UPR) in the endoplasmic reticulum (ER). The UPR pathway has two arms: (a) the ER membrane‐localized RNA splicing factor IRE1 is involved in the unconventional splicing of *bZIP60* pre‐mRNA, resulting in the expression of functional transcription factor; (b) the pair of ER membrane‐anchored TFs, bZIP17 and bZIP28, is released and relocated to the Golgi apparatus, cleaved by S1P and S2P proteases, and transported to the nucleus. In the nucleus, bZIP17/60 and bZIP28/60 dimers activate stress‐responsive gene expression on the ERSE/UPRE. Abbreviations: bZIP, basic leucine zipper TF; Ca^2+^, calcium cation; ER, endoplasmic reticulum; ERSE, ER stress‐response element; GA, Golgi apparatus; IRE1, inositol requiring enzyme 1α; PM, plasma membrane; ROS, reactive oxygen species; S1P, site‐1 protease; S2P, site‐2 protease; TFs, transcription factors; UPR, unfolded protein response; UPRE, unfolded protein response element.

The accumulation of misfolded or unfolded proteins in the ER at elevated temperatures activates the UPR, which is conserved amongst eukaryotic organisms (Fig. 2). In order to set the UPR in motion, plants use UPR sensors to monitor the protein folding status in the ER (Iwata & Koizumi, 2005; Liu *et al*., 2007; Liu & Howell, 2010). The UPR pathway was shown to be active in both vegetative and reproductive development, defence, bacterial and viral immunity (Bao & Howell, 2017). In vegetative tissues, there are two arms of the UPR pathway. First, ER membrane‐localized RNA splicing factor INOSITOL REQUIRING ENZYME 1 (IRE1), harbouring both protein kinase and RNase domains in its cytoplasmic C‐terminal portion, is involved in the unconventional splicing of *bZIP60* pre‐mRNA (Fig. 2). It results in the expression of functional transcription factor (TF) via the elimination of its transmembrane domain (Deng *et al*., 2011). The second UPR arm involves bZIP17 and bZIP28, a pair of ER membrane‐anchored TFs. They are typically retained in the ER membrane by their association with the luminal BiP protein. Following HS, they are released and relocated to the Golgi apparatus. There, bZIP17 and bZIP28 are cleaved by S1P and S2P proteases and, upon release, are transported to the nucleus where they activate stress‐responsive gene expression (Liu *et al*., 2007; Liu & Howell, 2010). When looking at plant reproductive development specifically, only the first arm of the UPR pathway has been shown to help to protect male gametophyte development from HS, even though the expression of genes active in both UPR arms were detected during pollen development (Fragkostefanakis *et al*., 2016a). Tissue profiling of *Arabidopsis* early and late flowers revealed distinct heat stress responses in vegetative and generative tissues, with genes participating in the UPR being enriched among heat‐upregulated reproductive tissue‐specific genes (S. S. Zhang *et al*., 2017). Moreover, in *Arabidopsis,* both the *ire1a/ire1b* double mutant and the bZIP60 single mutant showed male sterility at higher temperatures (Deng *et al*., 2016). Interestingly, two pollen‐expressed cytoplasm‐localized bZIP TFs, bZIP18 and bZIP52, were recently shown in *Arabidopsis* seedlings to accumulate in nuclei following HS. However, their re‐localization was triggered by dephosphorylation, probably at the serine residues within their conserved HXRXXS motifs (Wiese *et al*., 2021).

The accumulation of unfolded proteins in the cytoplasm elicits the UPR via heat shock protein (HSP)–heat shock factor (HSF) complexes (Bokszczanin *et al*., 2013). HsfA1 acts as a master regulator of the HS activation network in vegetative tissues (Mishra *et al*., 2002; Yoshida *et al*., 2011). Knockdown and multiple knockout mutants of *HsfA1* genes in *Arabidopsis* and tomato vegetative tissues resulted in the reduced induction of many HS‐responsive genes, which concurrently produced HS‐sensitive phenotypes (Mishra *et al*., 2002; Yoshida *et al*., 2011). Upon HS, HsfA1 directly activates the second important player, DEHYDRATION‐RESPONSIVE ELEMENT BINDING PROTEIN 2A (DREB2A) and several other TFs, including HsfA2 (Yoshida *et al*., 2011). DREB2A integrates heat‐ and drought‐stress responses by activating the respective sets of genes (Ohama *et al*., 2017).

In tomato pollen, HsfA2 regulates a subset of HS‐induced genes (including several HSPs) and acts as an essential co‐activator of HsfA1a during the HSR (Giorno *et al*., 2010; Fragkostefanakis *et al*., 2016b). Heat shock proteins play a key role in mitigating the effects of HS on plant metabolism, acting predominantly as molecular chaperones and protecting proteins from the harmful effects of stress (e.g. conformation changes, aggregation), thereby maintaining protein homeostasis during the stress period (Vierling, 1991; Kotak *et al*., 2007; Mishra & Grover, 2015). HsfA2 suppression reduced pollen viability and germination when HS was applied during the meiosis and microspore formation stages, but had no effect later on. This highlights the fact that HsfA2 is an important player in the priming process that sustains pollen thermotolerance during microsporogenesis (Fragkostefanakis *et al*., 2016b). The AtREN1 (RESTRICTED TO NUCLEOLUS1) protein, a close homologue of HsfA5, also contributes to pollen thermotolerance. This protein is explicitly targeted to the nucleolus and is likely to be involved in ribosomal RNA biogenesis or other nucleolar functions. *Atren1/‐* plants are defective in the HSR and produce a notably higher proportion of aberrant pollen grains (Reňák *et al*., 2014).

## The ‐omics approach to unravelling unknown aspects of the HSR

**Fig. 3:**
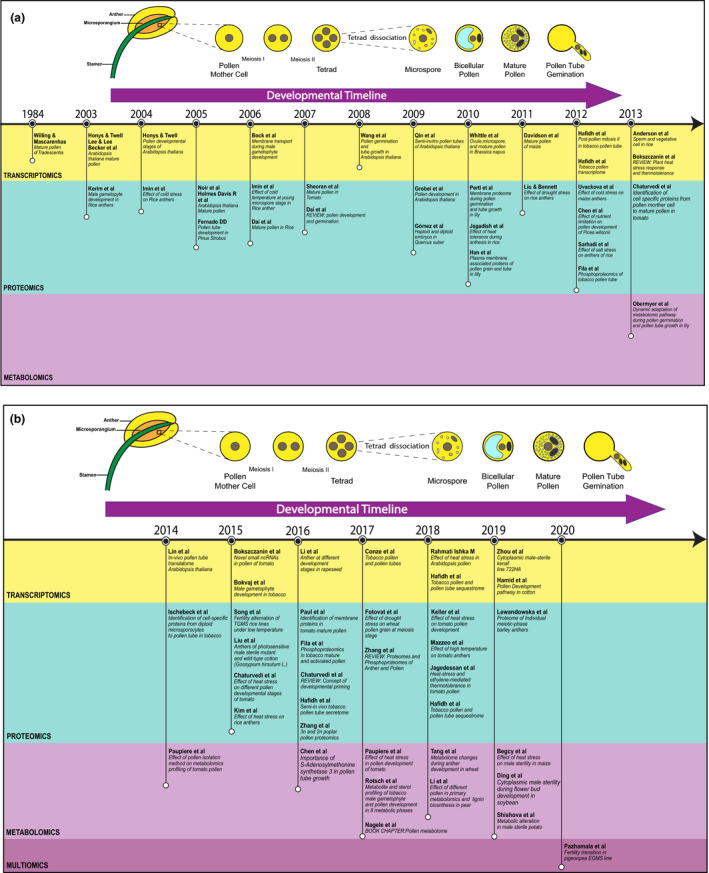
Timeline charting some of the important advances in male gametophyte (pollen) ‘‐omics’ studies based on developmental stages, cell types, techniques, and species. (a) Years 1984–2013; (b) Years 2014–2020.

To counter the negative effects brought on by HS, plants activate a HSR based on the initial stress, bringing about a hierarchical re‐programming of the transcriptome, proteome and metabolome (Mittler *et al*., 2012). With the advancement of isolation techniques over the years, researchers are now able to study multiple ‐omics in reproductive cells (e.g. pollen grains, sperm cells, egg cells, pollen tubes, Fig. 3) (Holmes‐Davis *et al*., 2005; Dai *et al*., 2006; Sheoran *et al*., 2007; Borges *et al*., 2008; Sheoran *et al*., 2009; Fíla *et al*., 2012; Chaturvedi *et al*., 2013; Obermeyer *et al*., 2013; Ischebeck *et al*., 2014; Chaturvedi *et al*., 2015; Fíla *et al*., 2016; Chaturvedi *et al*., 2016; Julca *et al*., 2020). In Fig. 3, we have assembled a summary of some of the crucial advances made in deciphering pollen development, under control and stress conditions, using ‐omics technologies. Similarly, Table 1 lists the stress response mechanisms elucidated for the male gametophytes of different species.

**Table 1:** Overview of the omics and classical studies published to understand stress response mechanism of male gametophyte in different plant species.

Order	Plant species	Temperature (°C)/duration of exposure (h/d)	Effect on pollen	References
Poales	*Brachypodium distachyon*	32°C	Decline in pollen viability, retention of pollen in anthers and pollen germination	Harsant *et al*. (2013)
		36°C	Abortion of microspores by the uninucleate stage, aberrations in tapetal development and degeneration	Harsant *et al*. (2013)
	*Hordeum vulgare*	30°C	Aberrations in tapetal development and degeneration	Abiko *et al*. (2005)
	*Oryza sativa*	33°C	Reduced fertility and seed set	Ziska *et al*. (1996)
	*Triticum aestivum*	> 5°C above ambient temperature	Reduction in pollen production and viability	Stone & Nicolas (1995)
		30°C/3 d	Tapetum degeneration	Saini *et al*. (1984)
	*Zea mays*	38°C	Affected pollen–stigma interactions	Mitchell & Petolino (1988)
		35/25°C	Irregular tetrads	Begcy *et al*. (2019)
Vitales	*Vitis vinifera*	> 35°C	Alternative splicing	Jiang *et al*. (2017)
Fabales	*Cicer arietinum*	35/20°C	Reduced pollen germination and tube growth	Devasirvatham *et al*. (2012)
		45/35°C	Decreases the concentration of soluble sugars in the anther walls of developing and mature pollen grains	Ismail & Hall (1999)
	*Phaseolus vulgaris*	32/27°C/1–5 d	Decreases the concentration of soluble sugars in the anther walls of developing and mature pollen grains	Suzuki *et al*. (2001)
	*Pisum sativum*	35°C/4–7 d	Reduced pollen viability and the proportion of ovules that received a pollen tube	Jiang *et al*. (2019)
	*Vigna unguiculata*	33/20°C	Aberrations in tapetal development and degeneration	Ahmed *et al*. (1992)
Rosales	*Pyrus bretschneideri*	Low temperature of 4°C	Inhibits pollen tube growth	Gao *et al*. (2014)
	*Rosa* sp.	36°C/48 h	Alterations in male meiotic chromosome behaviour resulting in meiotically restituted dyads and triads	Pecrix *et al*. (2011)
Brassicales	*Arabidopsis thaliana*	30–32°C	Alterations in cross‐over distribution and induction of male meiotic restitution	De Storme & Geelen (2020)
Malvales	*Gossypium hirsutum*	> 30°C	Pollen sterility and abortion	Ismail & Hall (1999)
Malpighiales	*Populus tremula*	38°C	Large spherical grains and pollen abortion	Wang *et al*. (2017)
Caryophyllales	*Fagopyrum esculentum*	30°C	Ovules more sensitive compared to pollen grains	Płażek *et al*. (2019)
Solanales	*Nicotiana tabacum*	Heat	Altered the structure of cytoskeletal network of pollen tubes	Parrotta *et al*. (2016)
	*Solanum lycopersicum*	43–45°C/2 h	Reduced viability	Muller & Rieu (2016)
		50°C/2 h	Decrease in germination rate	Firon *et al*. (2012), Jegadeesan *et al*. (2018)

### Heat stress response – re‐programming of the transcriptome

Transcriptome profiling provides a global snapshot of all RNA species (mRNA, tRNA, sRNA and microRNA) present in a sample at any given time point, which cannot be studied at the genomic level (Weckwerth *et al*., 2020). The Affymetrix ATH1 GeneChip has been the most widely used microarray for *Arabidopsis* cell type profiling of male and female reproductive lineages (Schmidt *et al*., 2012). Apart from microarrays, Next Generation Sequencing (NGS)‐based RNA sequencing (RNA‐seq) is now routinely used for transcriptional profiling. It has a broader dynamic range and higher sensitivity, and it offers whole‐genome coverage leading to the identification of unknown transcripts and novel splice variants (Schmidt *et al*., 2012; Loraine *et al*., 2013; Julca *et al*., 2020). The major advantage of RNA‐seq is the applicability to nonmodel species afforded by the continuous development of computational methods for data integration from various studies using different platforms and methods. Comparative transcriptomic analysis has revealed 5,365 genes that are differentially expressed in the heat‐stressed switchgrass (*Panicum virgatum)* cv. Alamo (Li *et al*., 2013). Moreover, HS has been shown to alter approximately 15% of the *Arabidopsis* pollen transcriptome when compared to control conditions (Rahmati Ishka *et al*., 2018). The earlier developmental stages were more sensitive to temperature fluctuations than the later stages (Raja *et al*., 2019), illustrating the limited capacity of the earlier stages in inducing a proper HSR.

**Fig. 4:**
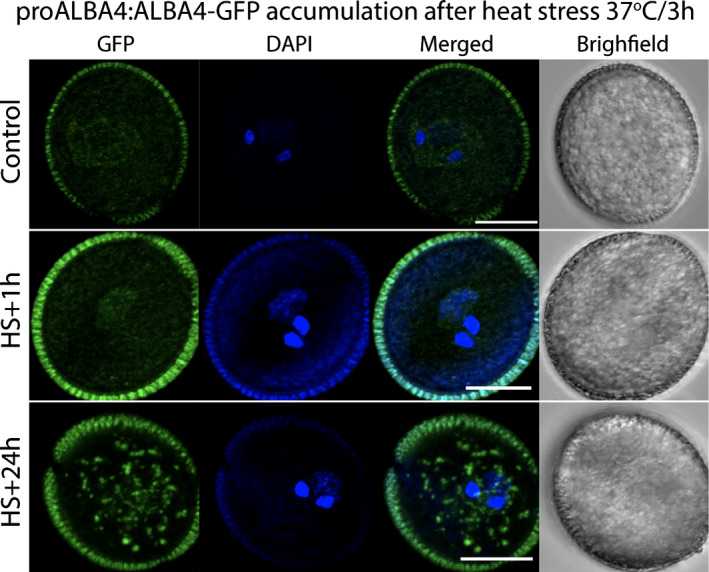
Effect of heat stress on the redistribution of ALBA‐family RNA‐binding protein ALBA4 (At1g76010) in pollen and its accumulation in large cytoplasmic granules 24 h after the heat stress (Náprstková *et al*., 2021). Arabidopsis plants cultivated at 22°C were heat‐stressed at 37°C for 3 h and then transferred back to their growth temperature. Mature pollen was collected 1 h and 24 h after the end of HS treatment and observed by bright field and fluorescent microscopy under control conditions (top row) and 1 h (middle row) and 24 h (bottom row) after the heat shock treatment (37°C for 3 h). Bars, 10 μm.

Although the majority of HS‐responsive genes are regulated at the transcriptional level, transcriptomic studies also revealed the stress‐related regulation of gene expression at post‐transcriptional levels, namely mRNA processing and translation. A genome‐wide study in tomato pollen revealed alternative splicing (AS) as a new regulatory level for genes with a constitutive expression pattern (Keller *et al*., 2017). Alternative splicing is also implicated in the HSR in the vegetative tissues of grape (*Vitis vinifera*) (Jiang *et al*., 2017) and cabbage (*Brassica oleracea*), where genes upregulated by HS showed an increase in AS events (Lee *et al*., 2018). These genes included HSFs and HSPs, with the authors Lee *et al*. (2018) suggesting a role for AS in HS adaptation. The relationship between transcriptional and translational regulation under diverse and combined stress conditions (drought, heat) across different tissues was assessed by comparing transcriptomic and translatomic data (Matsuura *et al*., 2010; Li *et al*., 2018; Poidevin *et al*., 2020). For example, *in vitro* germinated *Arabidopsis* pollen responded to severe stress conditions by upregulating heat shock genes in a similar manner to vegetative tissues. Ribosome profiling combined with RNA‐seq revealed high correlation between transcriptional and translational responses to high temperatures, while specific regulations at the translational level were also observed (Poidevin *et al*., 2020). Post‐transcriptional regulation and ribosome re‐arrangement during the HSR involves the redistribution of mRNA and RNA‐binding proteins between actively translating ribosomes and cytoplasmic mRNA granules. In plants, they comprise stress granules (SGs) and processing bodies (PBs), where mRNA is sequestered and/or processed during HS (Weber *et al*., 2008; Chantarachot & Bailey‐Serres, 2017; Kosmacz *et al*., 2019). Such HS‐dependent mRNA rearrangement has been observed in pollen, as exemplified by the re‐distribution and accumulation of pollen‐expressed RNA‐binding protein ALBA4 in cytoplasmic granules during long‐term HS (Fig. 4). These cytoplasmic granules probably represent SGs, as suggested by the co‐localization of ALBA4 with PABP3 in pollen, but direct evidence is lacking (Náprstková *et al*., 2021). The suggested presence of SGs in pollen upon HS (Billey *et al*., 2020; Náprstková *et al*., 2021) would distinguish them from proposed general mRNA‐storage compartments, processing bodies (Scarpin *et al*., 2017) and nontranslating monosomes (Hafidh *et al*., 2018; Urquidi Camacho *et al*., 2020).

Several studies employing chromatin immunoprecipitation demonstrated the involvement of epigenetic processes in the pollen HSR (Chen *et al*., 2016). For example, HS affects DNA methylation and the expression of methyltransferase genes (Solis *et al*., 2012), and the methylation of genes encoding HSPs (Migicovsky *et al*., 2014; McCue *et al*., 2015), and influences chromatin conformation (Lang‐Mladek *et al*., 2010; Pecinka & Mittelsten Scheid, 2012). The importance of small noncoding RNAs (sncRNAs) in the regulation of pollen development at both transcriptional and epigenetic levels has been well documented (Slotkin *et al*., 2009; Calarco *et al*., 2012). A study performed by Bokszczanin *et al*. (2015) identified a complex set of tomato pollen sncRNAs (miRNAs, tRNAs, and snoRNAs) which were affected by HS in a stage‐specific manner. Interestingly, gene ontology enrichment analysis revealed that most target genes of all expressed miRNAs were significantly enriched in protein binding, transcription, and serine/threonine kinase activity. An outstanding result of this study demonstrated that tRNAs responded to HS not uniformly, but rather in an amino‐acid dependent manner, with the most dramatic response observed at later developmental stages (Bokszczanin *et al*., 2015).

### Heat stress response – re‐programming of the proteome/restoring protein homeostasis

The cellular proteome does not fully reflect the transcriptome, especially in systems with high levels of translational regulation, such as the male gametophyte. Therefore, it is necessary to complement transcriptomics with translatomic and proteomic data to get a more realistic insight (Fíla *et al*., 2017). Compared to the transcriptomic approach, proteomic analyses require larger amounts of starting material, which represents a major limitation of using this technique for the younger developing stages of pollen (e.g. pollen mother cells, tetrads, microspores). For example, 40 µg of proteins isolated from different developmental stages of tomato pollen is required for a standard proteomic study (Chaturvedi *et al*., 2013; Chaturvedi *et al*., 2015). The availability of proteomic data demonstrated a low level of correlation between transcript and protein levels in the tomato pollen HSR (Jegadeesan *et al*., 2018; Keller & Simm, 2018). Since the transcriptome and translatome correlated well during *Arabidopsis* pollen HSR (Poidevin *et al*., 2020), post‐translational levels also seem to be involved. Interestingly, HS treatment of tomato pollen showed not only the uncoupling of the pollen HSR at transcriptional and post‐transcriptional levels, but also the differential upregulation of an unusually high proportion of pollen‐specific transcripts and proteins (Jegadeesan *et al*., 2018; Keller & Simm, 2018). These qualitative and quantitative differences, which only partly reflected the generally higher representation of specific genes in the male gametophyte (Honys & Twell, 2004; Rutley & Twell, 2015), should be attributed also to the sensitivity of the methods employed. Thus, in order to understand the pollen HSR in more detail, the integration of transcriptomic and proteomic datasets is essential.

As mentioned previously, HSFs regulate the expression of a diverse group of HSPs by recognizing heat stress elements (HSE) in their promoters, repetitive patterns of palindromic binding motifs (5′‐AGAAnnTTCT‐3′) upstream of the TATA box (Scharf *et al*., 2012). The majority of the transcriptome fraction induced by HS, however, encodes a diverse set of genes, not only HSPs but also genes encoding HSFs and metabolic enzymes, such as *INOSITOL‐3‐PHOSPHATE SYNTHASE2* (*IPS2*) and *GALACTINOL SYNTHASE1* (*GOLS1*) (Liu *et al*., 2011).

Several eukaryotic translation initiation factors (eIFs) are affected by stress treatment, and a few of them have even been shown to affect the stress response. Heterologous overexpression of several eIFs subunits in plants improved their tolerances to specific abiotic stresses. For example, transgenic *Arabidopsis* plants expressing *Rosa chinensis RceIF5A*, showed improved leaf thermotolerance and an increased resistance towards oxidative and osmotic stresses (Xu *et al*., 2011). Conversely, the *Arabidopsis hot3* mutant, with impaired *eIF5B1* expression, was unable to acquire tolerance to elevated temperatures (Hong & Vierling, 2000) and showed delayed recovery of translation apparatus following heat stress, accompanied by reduced translation efficiency of a subset of stress‐protective proteins (L. Zhang *et al*., 2017). The comparison of weak *hot3‐1* and severe *hot3‐2* alleles suggested the mechanism, whereby disrupting specific eIF5B interactions on the ribosome can affect translation, directly or indirectly (L. Zhang *et al*., 2017). Selective regulation of translation under heat stress conforms to the selective regulation of amino‐acid‐specific tRNAs under heat stress in tomato (Bokszczanin *et al*., 2015).

### Heat stress response – restoring metabolite homeostasis

The plant metabolome is highly complex, as it emerges from both primary and secondary metabolism. It is estimated that there are *c*. 200 000 different metabolites present in plants (Sheth & Thaker, 2014). Unfortunately, the major limitation in high throughput metabolome profiling is the lack of a unified method that allows comprehensive measurements in terms of detection, quality, quantity and spatio‐temporal resolution. This is primarily because each metabolite differs in terms of concentration and chemical and analytical properties (Ghatak *et al*., 2018). A major analytical challenge as it relates to pollen is the removal of the pollenkitt and the other hydrophobic compounds on the pollen coat, which hinder the detection and identification of various metabolites (Obermeyer *et al*., 2013).

Heat stress has been shown to bring about metabolic imbalance (Kaplan *et al*., 2004). In this respect, the accumulation of reactive oxygen species (ROS) is a reliable marker of stress (Fig. 2, see above). An increase in ROS levels in stressed pollen correlates with a 60% reduction in the *Arabidopsis* and tomato pollen germination rate (Luria *et al*., 2019). With regards to the stress response, several studies have indicated a strong interaction between sugar‐ and abscisic acid (ABA)‐mediated signaling pathways (Gibson, 2004). Abscisic acid was shown to repress the expression of anther cell wall‐associated invertase (responsible for sucrose cleavage) in wheat, which resulted in a perturbation of sugar metabolism in developing spores (Ji *et al*., 2011). The application of exogenous auxin can enhance heat tolerance in rice reproductive organs (Zhang *et al*., 2018) and reduce the occurrence of male sterility. A similar phenomenon was observed in pigeonpea (*Cajanus cajan*), where the exogenous application of auxin could induce the expression of auxin transport proteins in pollen, which are required for cell wall development and nutritive supply under unfavourable conditions (Pazhamala *et al*., 2020). In this respect, temperature‐dependent decreases in auxin levels hamper normal cell wall development and contribute to male sterility in pigeonpea (Pazhamala *et al*., 2020). Other hormones have also been implicated in the stress response. For example, it has been proposed that the regulatory interaction between jasmonic acid and carbohydrate metabolism controls water transport into the anther, perhaps via induction of the *AtSUC1* gene (Ishiguro *et al*., 2001).

Pollen development and germination comprise multiple steps of metabolic regulation, leading to significant metabolome dynamics (Nägele *et al*., 2017). Comprehensive profiling of metabolites under stress conditions and putting the obtained results in perspective with integrated transcriptomic and proteomic datasets, will be another step forward in producing stress‐tolerant crops.

## Thermotolerance mechanisms – pollen perspectives

The adverse effects brought on by HS can be circumvented to some extent when plants undergo a ‘pre‐conditioning treatment’. Basal thermotolerance refers to a plant’s competence in withstanding nonlethal heat stress (e.g. acute heat stress; 36–45°C applied for 1–3 h) (Chaturvedi *et al*., 2015; Fragkostefanakis *et al*., 2015; Mesihovic *et al*., 2016). When a mild stress treatment is followed by a short recovery phase (i.e. pre‐conditioning), acquired thermotolerance is induced, which enables plants to withstand usually lethal heat stress. This phenomenon can be attributed to the ability of plant cells to store proteins which can enhance their tolerance to high temperatures that might otherwise be lethal (Larkindale & Vierling, 2008). For example, tomato Micro‐tom plants showed a dramatic decrease in their germination rate when subjected to 50°C temperature conditions for 2 h (Firon *et al*., 2012; Jegadeesan *et al*., 2018). However, a pre‐conditioning treatment (32°C for 1 h) followed by a recovery phase (25°C for 1 h), resulted in enhanced tolerance to 50°C for 2 h compared to plants that had not been pre‐conditioned (Firon *et al*., 2012). Such pre‐conditioning during the reproductive phase is not specific to pollen – it also affects seed development. A comparison between a gradual temperature increase and an acute 40°C stress treatment showed that the gradual acclimation enhanced seed thermotolerance (Stone & Nicolas, 1995). It not only confirmed the ability of reproductive tissues to acquire thermotolerance by applying a gradual and incrementally increasing HS treatment, but also highlighted the importance of using natural stress conditions in thermotolerance screens. Further studies on tomato have demonstrated that hormonal pre‐treatment is also beneficial for pollen fitness in stress conditions. Pre‐treating tomato plants with ethylene (ethephon) before HS caused a significant increase in pollen quality following a HS treatment (Jegadeesan *et al*., 2018). The induced pollen proteome of ethephon‐treated plants showed that there is an abundance of proteins that are implicated in the maintenance of the cellular redox state, which likely minimizes the effect of HS (Jegadeesan *et al*., 2018).

Pollen thermotolerance is an economically increasingly important trait for breeding; hence, it is necessary to develop suitable protocols which will allow high throughput evaluation of pollen quality in changing environmental conditions, together with the establishment of an appropriate pre‐treatment methodology that is applicable to crops growing in endangered areas.

## Understanding natural variation in pollen thermotolerance

Exploring natural variation may offer insight into the genetic background of heat stress tolerance and can maintain diversity, which is favourable for breeding (Grandillo *et al*., 2007; Weckwerth *et al*., 2020). This also applies to heat tolerance of reproductive tissues, but so far there is minimal screening for variation in heat sensitivity in plant species with a reproductive system that is considered to be thermotolerant. There seem to be at least two significant reasons for this. Firstly, germplasm screening is limited to fruit sets. Fruit set is a very complex trait which is always accompanied by sub traits. For example, it has been shown that decreases in number of tomato fruits under long‐term mildly elevated temperatures correlated with lower pollen viability. (Pressman *et al*., 2002; Pressman *et al*., 2006). Therefore, investigating the individual traits separately can provide a better understanding of the genetic basis of reproductive thermotolerance under heat stress. Secondly, the choice of germplasm used for screening also plays an important role. It directly indicates the dimension of the genetic base used for screening; for example, several studies only use germplasm that consists mostly of cultivable tomato genotypes for thermotolerance screening (Dane *et al*., 1991; Grilli *et al*., 2007). Driedonks *et al*. (2018) investigated thermotolerance in reproductive tissues of 64 accessions across 13 wild species and seven tomato cultivars. These included a subset that showed satisfying reproductive thermotolerance under control conditions and following exposure to long‐term mild heat (LTMH). In this study, the LA1630 genotype showed the best performance in terms of pollen viability under LTMH, which demonstrates that the screening of wild germplasm can also enrich our knowledge of reproductive thermotolerance. In addition, the authors concluded that pollen viability and the quantity of pollen grains produced per flower are two main variables which can be used in further analysis, and pollen viability is an adaptive variable which depends on local conditions. However, it has also been proposed that further QTL analysis could be used to identify phenotypic traits under LTMH stress (Driedonks *et al*., 2018). Reproductive success depends on multiple traits; to identify these, and to significantly improve tolerance of the effects of heat stress on reproduction, more studies are needed at the genome‐wide level. The identified traits can be further transferred or used in marker‐assisted breeding for the development of resilient crops through genetic modification methods.

## Concluding remarks and considerations for going forward

With regards to abiotic stress, temperature fluctuations are seldom an isolated event. Heat stress often coincides with drought stress and higher light intensities (Raja *et al*., 2019). Thus, how pollen responds to multiple co‐occurring stresses may differ greatly compared to its response to heat stress alone. This has been demonstrated for tobacco. In an attempt to simulate realistic environmental conditions, Rizhsky *et al*. (2002) subjected tobacco plants to either a single stress – heat or drought – or to both simultaneously. Using transcriptomics, the authors were able to show that plants responded differently when both stresses were applied at once, since the genes upregulated in the latter were not affected when a single stress was applied. Hence, experiments need to be designed that mimic real‐life conditions to yield information that breeders can use to generate crops tolerant to these scenarios. The ‐omics tools can be of great use in this regard, since they have already proved themselves fundamental in unravelling the mechanisms male gametophytes employ in response to heat stress. However, the majority of articles published on heat stress and gametophyte development have been based on transcriptomics and proteomics, with contributions from metabolomics and other more specialized ‐omics (e.g. lipidomics) still lagging (Paupière *et al*., 2017b; Mazzeo *et al*., 2018). Finally, the large quantities of data generated from the different ‐omics experiments need to be integrated, to provide plant breeders with targeted information on genes or alleles they can use to improve germplasm and to develop tolerant lines. In this review, we provided an overview of the stress response in the male gametophyte and highlighted pollen thermotolerance mechanisms. Furthermore, we elaborated on reproductive organ defects, regulation of gene expression, and the maintenance of protein and metabolite homeostasis under stress conditions, while summarizing the three central ‐omics approaches that deepen our understanding of the stress response mechanism.

## Author contributions

PC and DH conceived the study; PC, AJW, AG, LZD, WW and DH drafted the manuscript; PC, AG, and DH designed the figures; and LZD prepared the data table. All authors have revised and approved the final version of the manuscript.
